# Employing complementary fractionation‐based N‐terminomics approaches enhances the identification of legumain cleavage events in naïve and inflamed colon tissue

**DOI:** 10.1002/pro.70186

**Published:** 2025-09-19

**Authors:** Alexander R. Ziegler, Benjamin L. Parker, Nichollas E. Scott, Laura E. Edgington‐Mitchell

**Affiliations:** ^1^ Department of Biochemistry and Pharmacology Bio21 Molecular Science and Biotechnology Institute, The University of Melbourne Parkville Victoria Australia; ^2^ Department of Anatomy and Physiology, Medical Building 181 The University of Melbourne Parkville Victoria Australia; ^3^ Department of Microbiology and Immunology Peter Doherty Institute, The University of Melbourne Parkville Victoria Australia

**Keywords:** basic reverse‐phase, colitis, FAIMS, fractionation, HUNTER, legumain, N‐terminomics, protease, substrates, TMTpro

## Abstract

The mammalian lysosomal protease legumain is often dysregulated in pathophysiological conditions including inflammation, neurodegeneration, and cancer, yet its proteolytic targets are poorly defined. To profile protease substrates, degradomics techniques typically employ enrichment strategies to select for sub‐stoichiometric and low‐abundance peptides generated by proteolytic cleavage. However, recent advancements in degradomics techniques have revealed N‐termini enrichment can be circumvented if peptide‐based fractionation is employed, enabling simultaneous proteome and N‐terminome analysis. Herein, we compare the previously published enrichment‐free N‐terminomics approach using high‐field asymmetric waveform ion mobility spectrometry (FAIMS) to offline basic reverse‐phase (bRP) fractionation to assess the complementarity of these fractionation methods for simultaneous proteomic and degradomic analyses. While at the protein level FAIMS and bRP provide access to overlapping proteomic coverage, at the N‐terminus level each fractionation technique reveals unique cleavage information. Combining data from the two fractionation approaches revealed 6499 N‐terminal peptides with N‐terminal TMTpro labeling, allowing the identification of cleavage events modulated in the context of legumain deficiency in naïve murine colons and during dextran sulfate sodium (DSS)‐induced colitis. Among these N‐termini, we identify 35 putative legumain substrates in naïve and 41 in the DSS‐treated colons, supporting a role for legumain in both pro‐inflammatory and physiological conditions. Use of an additional negative selection method, High‐efficiency Undecanal‐based N‐Termini EnRichment (HUNTER), further supplements this list of identified legumain substrates. Combined, this study identifies multiple putative substrates of legumain in healthy and inflamed murine colons as well as demonstrates the utility of using complementary fractionation approaches for degradomics studies.

## 1 INTRODUCTION

Proteolysis is an irreversible post‐translational modification (PTM) catalyzed by proteases and is crucial for regulating proteostasis, either through degradation of proteins or generation of stable cleavage products that contribute to biological processes (López‐Otín & Bond, 2008) including cell proliferation (Scher, 1987), apoptosis (Moffitt et al., 2010), cell migration (Elzer et al., 2008), and immune signaling (Conus & Simon, 2010). Dysregulation of proteolytic activity is associated with a range of pathophysiological conditions, including inflammation (Schuliga, 2015), neurodegeneration (Rai et al., 2022), and cancer (Song et al., 2021). Defining cleavage events is essential for improving our understanding of protease‐mediated pathophysiological processes and how these conditions may be controlled.

Several mass spectrometry‐based approaches to identify protease‐regulated cleavage events have been developed as previously reviewed (Grozdanić et al., 2019; Mintoo et al., 2021). These proteomics‐based methods involve the detection of either C‐ or N‐terminal peptides resulting from proteolytic cleavage. Due to the high efficiency of amine labeling, N‐termini‐focused techniques collectively known as N‐terminomics dominate the field (Canbay & auf dem Keller, 2021; Wang, Main, et al., 2021). Within proteome samples, native and protease‐generated (neo) N‐terminal peptides comprise only a small percentage of the total proteome, requiring the enrichment of this subpopulation to allow their identification using early N‐terminomic pipelines (Kaushal & Lee, 2021). This requirement drove the use of affinity enrichment of N‐termini with positive enrichment strategies such as chemical enrichment of protein substrates (CHOPS) as well as subtiligase‐based labeling techniques, facilitating the enrichment of these peptides prior to analysis by liquid chromatography tandem mass spectrometry (LC–MS/MS) (Bridge et al., 2024; Griswold et al., 2019; Weeks et al., 2021). Conversely, negative selection methods have also been developed, which leverage protein level dimethylation (Doucet & Overall, 2011) or N‐hydroxysuccinimide‐based isobaric labeling such as tandem mass tags (TMT) (Kalogeropoulos et al., 2023) to chemically block N‐termini allowing internal peptides generated during sample proteolysis to be depleted from the samples based on their reactive N‐termini (Prudova et al., 2010). Negative selection approaches such as terminal amine isotopic labeling of substrates (TAILS) employ hyperbranched polyglycerol‐aldehyde (HPG‐ALD) polymers to capture internal peptides by binding unlabeled N‐termini (Kleifeld et al., 2010), whereas hydrophobic tagging‐assisted N‐termini enrichment (HYTANE) utilizes a bis‐hexadecyl chain tag and reverse‐phase chromatography to remove internal peptides based on their increased hydrophobicity (Chen et al., 2016). Similarly, High‐efficiency Undecanal‐assisted N‐Termini EnRichment (HUNTER) utilizes the hydrophobic undecanal tag to allow the depletion of internal peptides enabling the identification of N‐terminal peptides (Weng et al., 2019). Compared to other negative selection methods such as TAILS, which employ specialized polymers, these hydrophobic‐based methods employ readily available commercial reagents including reverse‐phase chromatography material such as C_18_ (Zamboulis et al., 2019). Moreover, HUNTER has been optimized for limited starting amounts (Demir et al., 2022), making it an accessible and attractive workflow for protease substrate profiling. Despite the utility of these enrichment‐based methods, recent advances in bioinformatic processing of proteomics data have also allowed for enrichment‐free N‐terminal peptide identification by incorporating semi‐specific cleavage parameters (Bell & Overall, 2023; Cosenza‐Contreras et al., 2024). Overall, these methods have facilitated the identification of protease cleavage events.

Orthogonal multi‐dimensional peptide fractionation is widely used in proteomic studies to reduce sample complexity and improve proteomic depth (Martinez‐Val et al., 2022; Mostovenko et al., 2013; Washburn et al., 2001). We recently demonstrated that online high‐field asymmetric waveform ion mobility spectrometry (FAIMS) fractionation not only improves proteomic depth but also allows enrichment‐free N‐terminomics comparable to published N‐terminomic enrichment techniques (Ziegler, Dufour, et al., 2024). While FAIMS is increasingly used to enable online fractionation of limited sample amounts (Cong et al., 2020) and the enrichment of peptide subsets such as glycopeptides (Ahmad Izaham et al., 2021), the performance of FAIMS has been noted to be more limited compared to offline fractionation approaches (Bekker‐Jensen et al., 2020). Over the last 20 years, the use of multiple offline chromatography approaches has been explored for in‐depth proteomic analysis, including ion exchange (Washburn et al., 2001; Xue et al., 2018), size exclusion (Bai et al., 2023), and reverse‐phase chromatography (Gilar et al., 2005). Of these approaches, basic reverse‐phase chromatography (bRP) has emerged as a popular approach due to its high degree of orthogonality to acidic reverse‐phase chromatography and high resolving power to enable deep proteome coverage (Bekker‐Jensen et al., 2017). These advantages allow bRP fractionation to detect low‐abundance peptides (Cao et al., 2012), such as phosphorylated peptides (Batth et al., 2014) while still being compatible with low sample input amounts (Kulak et al., 2017; Zurawska et al., 2023). Thus, we hypothesized that bRP fractionation may be ideally suited for improving the proteomic depth for simultaneous N‐terminome/proteome analysis.

Legumain is a lysosomal cysteine protease with unique asparaginyl endopeptidase (AEP) activity and contributes to various cellular functions such as lysosomal protein turnover, renal homeostasis, and immune signaling (Dall & Brandstetter, 2016; Miller et al., 2011). Dysregulation of its proteolytic activity is often associated with pathophysiological conditions such as inflammation (Edgington‐Mitchell et al., 2016; Tu et al., 2021); however, the mechanisms by which it propagates pathogenesis and the full extent of its substrates are largely unknown. We recently applied FAIMS‐facilitated N‐terminomics for the analysis of legumain‐deficient (*Lgmn* ^*−/−*^) and wild‐type (WT) murine colon tissues to assess the proteolytic contribution of legumain in both naïve and inflamed conditions (Ziegler, Anderson, et al., 2024). Building on this work, this study aimed to investigate the complementarity of online FAIMS fractionation and offline bRP fractionation for N‐terminome analysis. Utilizing TMTpro labeling to allow sample multiplexing (Wang, Kavdia, et al., 2020), we have assessed the depth and complementarity of N‐terminomic analyses following either online FAIMS or offline bRP fractionation. While both approaches allow deep proteomic coverage, the identification of unique N‐terminal peptides with bRP and the discovery of novel putative legumain substrates not previously detected in our FAIMS studies support the value of complementary fractionation approaches for N‐terminomic analysis. Additionally, using HUNTER, we validated the detection of N‐termini following fractionation, as well as identified additional N‐termini which were not detected using either bRP or FAIMS fractionation, further expanding the putative targets of legumain cleavage. Overall, this work demonstrates the analytical value of combining N‐terminomics approaches to improve the characterization of protease targets.

## 2 RESULTS

### 2.1 FAIMS and bRP fractionation provide complementary coverage of both the proteome and N‐terminome

**FIGURE 1:**
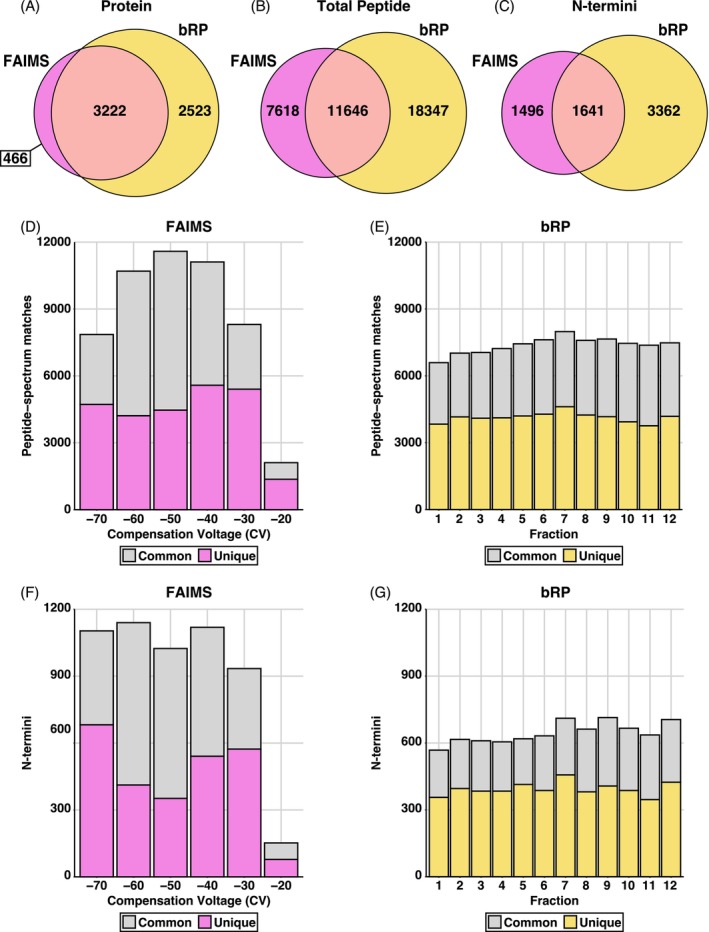
FAIMS and bRP fractionation produce complementary quantifications, which broaden the proteome coverage. (a)–(c). Overlap of unique protein (a), peptide (b), and N‐termini (c) identifications between the FAIMS and bRP fractionation workflows (*n* = 4/group). (d). Proportion of peptide‐spectrum matches identified in a single fraction (pink) or multiple fractions (gray) for FAIMS‐fractionated samples. Fractions were collected by altering the compensation voltage (CV) from −70 to −20 with 10 V intervals. (e). Proportion of peptide‐spectrum matches identified in a single fraction (yellow) or multiple fractions (gray) for basic reverse‐phase (bRP) fractionated samples. A total of 48 fractions were collected and concatenated in 12 fractions for analysis. (f), (g). Proportion of quantified N‐termini identified in a single fraction (pink/yellow) or multiple fractions (gray) for FAIMS (f) or bRP (g) fractionated samples.

We recently reported the use of FAIMS‐facilitated N‐terminomics analysis to assess the impact of legumain (Ziegler, Anderson, et al., 2024; Ziegler, Dufour, et al., 2024). While effective, it has been noted that FAIMS and bRP fractionation can provide complementary insights into the proteome (Klaeger et al., 2021). In light of this, we sought to benchmark the performance of the previously reported FAIMS‐fractionated method to an offline bRP fractionation approach for enrichment‐free N‐terminomics analysis. Using 16‐plex TMTpro labeling and quantitation, we compared healthy (naïve) and dextran sulfate sodium‐treated (DSS) colons of WT and legumain knockout (*Lgmn* ^*−/−*^) mice (Figure S1). Across both FAIMS and bRP fractionated samples, labeled pools demonstrate that all TMTpro channels possess comparable average intensities and dynamic ranges (Figure S2a, b) with a TMTpro labeling efficacy of ~90% (Figure S2c, f). Our previously reported FAIMS‐fractionated data identified a total of 3688 proteins, 19,264 peptides, and 3334 TMTpro‐labeled N‐terminal peptides (Ziegler, Anderson, et al., 2024). In comparison, bRP‐fractionated samples detected 5745 proteins and 29,993 peptides, including 5401 N‐termini (Tables S1 and S3). Between the FAIMS and bRP datasets, we observed a total of 466 unique protein and 7618 unique peptide identifications with FAIMS, while bRP identified 2523 unique proteins and 18,347 unique peptides (Figure 1a, b). Following filtering of the identified peptides for N‐terminal TMTpro labeling, 1496 N‐termini/cleavage sites were uniquely observed in the FAIMS dataset, while an additional 3362 N‐termini were identified using bRP (Figure 1c). These datasets exhibited substantial overlap, with 3222 proteins, 11,646 peptides, and 1641 N‐termini observed in both experiments (Figure 1a–c). Across FAIMS and bRP fractions, we observed a total of 75,342 peptide‐spectrum matches (PSMs) which were detected within only one fraction in the respective experiment, with the bRP fractions overall contributing 49,592 (>65%) of these unique PSMs (Figure 1d, e). Despite more unique peptides detected using bRP, on average, the FAIMS approach identified slightly more unique PSMs per fraction (4292 PSMs) than bRP (4133 average per fraction). Similarly, we observed a total of 7361 N‐termini identified in only one of the fractions within the respective experiment, with the majority (4723, >64%) of these originating from the bRP experiment (Figure 1f, g). While bRP identified more unique N‐termini per fraction, FAIMS again detected more unique N‐termini on average per fraction (440 in FAIMS vs. 394 in bRP). Between the two fractionation methods, the observed peptides and N‐termini overall showed no significant differences in their peptide properties, suggesting no clear bias in the selection for peptides despite the complementarity of these approaches (Figures S3 and S4). Combined, these data demonstrate that FAIMS and bRP provide complementary insights into both the proteome and N‐terminome.

### 2.2 Loss of legumain leads to alteration in the response to inflammation within the colon

**FIGURE 2:**
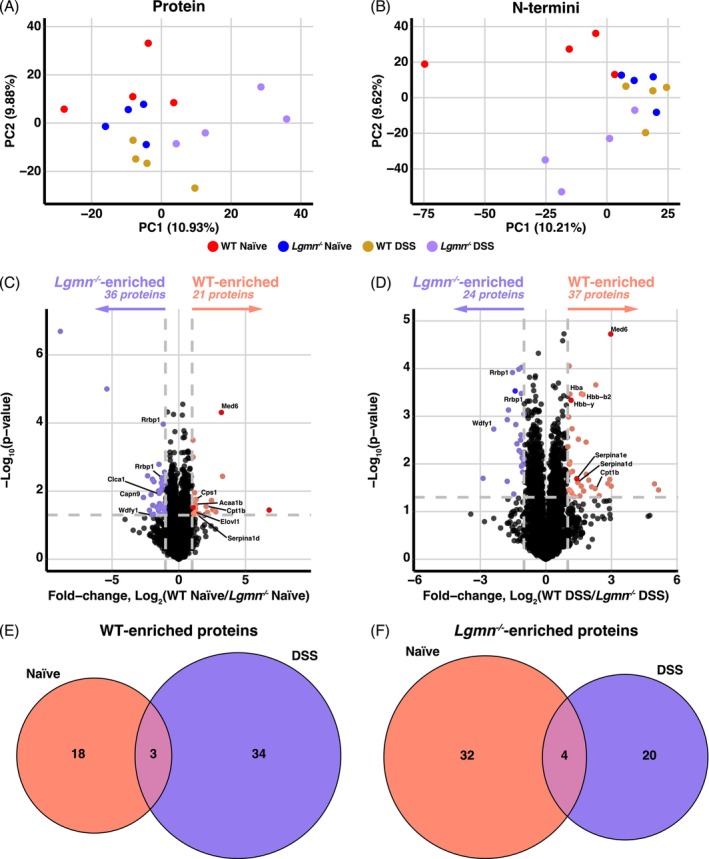
Legumain induces alterations to the proteome in both healthy and inflamed colons. Unique identifications in FAIMS‐fractionated samples were supplemented to the bRP identifications for combined analysis. (a), (b). Principal component analysis (PCA) of proteins (a) and N‐termini (b) identified by proteomics and N‐terminomics analyses following FAIMS and basic reverse‐phase (bRP) fractionation was performed based on biological replicates (*n* = 4/group). (c), (d). Following data analysis in FragPipe (v.22.0) and statistics in Perseus, proteins identified in either naïve (c) or DSS‐treated (d) colons were subjected to a student's two‐sample *t*‐test and visualized by volcano plot. A Log_2_(WT/*Lgmn* ^*−/−*^)>|1| and ‐Log_10_(*p*‐value)>1.3 were considered statistically significant (*n* = 4/group). (e), (f) Overlap of significantly changing proteins between naïve and DSS treatments in the WT (e) and *Lgmn* ^*−/−*^ (f) colon tissue.

To assess the impact of legumain on the global proteome in both the naïve and inflamed colon, we compared the proteomes of WT and legumain‐deficient (*Lgmn* ^*−/−*^) mice. Principal component analyses (PCA) at the protein and N‐termini levels revealed clustering associated with biological groups (Figure 2a, b) according to both genotype and treatment.

Compared to FAIMS, the number of protein quantifications following bRP fractionation was increased, such that 5745 proteins were quantified across the 16 replicates (Table S1). Considering FAIMS and bRP analyses were performed using identical samples, we analyzed the data in which proteins detected solely by the FAIMS approach were combined with the total bRP quantifications (Table S2). This included 466 proteins, for a total of 6211 proteins. Of these, 21 proteins in the naïve cohort were upregulated in WT mice, while 36 were downregulated (Figure 2c). These included significant increases in the total abundance of carbamoyl‐phosphate synthase (Cps1, Q8C196) and proteins involved in fatty acid metabolism such as 3‐ketoacyl‐CoA thiolase B (Acaa1b, Q8VCH0), carnitine O‐palmitoyltransferase 1 (Cpt1b, Q924X2), and very long chain fatty acid elongase 1 (Elovl1, Q9JLJ5) in WT mice, which may be crucial in the regulation of gut homeostasis and barrier function (Li et al., 2024). In the absence of legumain, both the calcium‐dependent cysteine protease calpain‐9 (Capn9, Q9D805) and the calcium‐activated chloride channel regulator 1 (Clca1, Q9D7Z6) were increased.

In inflamed colons, 37 proteins were increased in the WT mice and 24 in *Lgmn* ^*−/−*^ mice (Figure 2d). Of the WT‐enriched proteins, three were also enriched in naïve colons, including Cpt1b, Med6, and Serpina1d (Figure 2e), while the remaining 34 upregulated proteins were only significant in the inflamed colon and may represent proteins affected by the increased legumain activity associated with colitis. Similarly, in the absence of legumain, 24 proteins were significantly increased in abundance, and four of these were consistent with the naïve colon (Figure 2f). These four overlapping proteins included two different isoforms of ribosome‐binding protein 1 (Rrbp1, Q99PL5‐1/2), WD repeat and FYVE domain‐containing protein 1 (Wdfy1, E9Q4P1), and MLV‐related proviral Env polyprotein (P10404). A range of hemoglobin subunits (Hbb‐b2, Hbb‐y, and Hba) also showed an inflammation‐dependent increase in protein abundance in the presence of legumain (Figure 2d), potentially suggesting worsening of inflammatory conditions. Together, these data highlight the effects of legumain on the proteome of both healthy and inflamed colons and exemplify the utility of combining complementary approaches for deeper proteome coverage.

### 2.3 Complementary fractionation methods provide unique insights into novel legumain substrates

**FIGURE 3:**
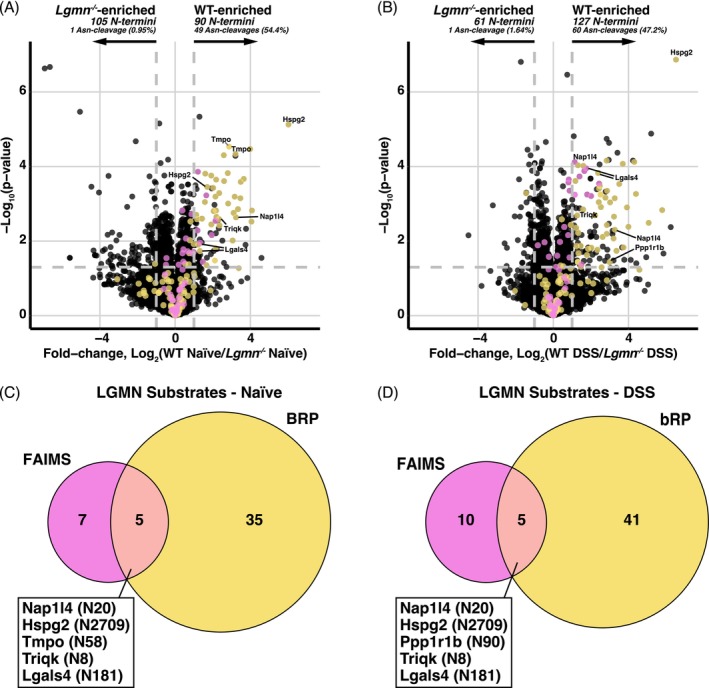
N‐terminomics analysis reveals legumain‐specific changes in both normal and inflamed colon tissue. (a), (b) Following data analysis in FragPipe (v.22.0) and statistics in Perseus, N‐termini were filtered from the total peptide identifications by filtering for N‐terminal TMTpro labeling. N‐termini identified in either naïve (a) or DSS‐treated (b) colons were subjected to a student's two‐sample *t*‐test and visualized by volcano plot. A Log_2_(WT/*Lgmn* ^*−/−*^) > |1| and ‐Log_10_(*p*‐value) >1.3 were considered as statistically significant (*n* = 4/group). N‐termini arising from cleavage following an asparagine residue are highlighted in yellow if identified using bRP or in pink if identified uniquely in FAIMS‐fractionated samples. (c), (d) Overlap of putative legumain substrates identified in naïve (c) and DSS (d) colon tissue between FAIMS‐fractionated (pink) and bRP‐fractionated (yellow) samples. The gene name and cleavage site of the overlapping identifications are indicated.

We next bioinformatically filtered TMTpro‐labeled N‐termini to distinguish native and protease‐generated (neo) N‐termini. We identified 5401 N‐termini with ≥3/4 quantifications in at least one of the groups using the bRP approach (Table S3). In line with our proteomic analysis, we combined the N‐termini uniquely identified by FAIMS with bRP, generating a total of 6881 unique N‐termini (Table S4). Among the detected N‐termini, several were significantly altered between the WT and legumain‐deficient (*Lgmn* ^*−/−*^) mice (Figure 3a, b), representing proteolytic changes either directly or indirectly due to legumain. To determine which of these substrates could be directly cleaved by legumain, we mined the dataset for N‐termini following asparaginyl cleavages. Of the 90 WT‐enriched N‐termini, 49 (54.4%) resulted from asparaginyl cleavage in the naïve colon (Figure 3a). In DSS‐treated mice, asparaginyl cleavage events made up 60 of 127 (47.2%) WT‐enriched N‐termini (Figure 3b). Together, these indicate putative legumain substrates within the naïve and inflamed colon, respectively. As expected, few cleavages following asparagine residues were enriched in the *Lgmn* ^*−/−*^ colon, consistent with the unique ability of legumain to act as an asparaginyl endopeptidase (Figure 3a, b).

In both naïve and inflamed conditions, cleavage of basement membrane‐specific heparan sulfate proteoglycan core protein (Hspg2) at ^2709^Asn↓^2710^Leu was the most enriched N‐terminus. Comparing the individual cleavage sites identified between the FAIMS and bRP fractionation workflows, we observed five cleavage sites common to naïve and inflamed colons (Figure 3c, d). In both naïve and inflamed tissues, these included cleavages of nucleosome assembly protein 1‐like 4 (Nap1l4) at ^20^Asn↓^21^Ala, basement membrane‐specific heparan sulfate proteoglycan core protein (Hspg2) at ^2709^Asn↓^2710^Leu, triple QxxK/R motif‐containing protein (Triqk) at ^8^Asn↓^9^Thr, and galectin‐4 (Lgals4) at ^181^Asn↓^182^Thr. Lamina‐associated polypeptide 2 (Tmpo) cleavage at ^58^Asn↓^59^Ser was also seen in the naïve colon, consistent with previous results in the spleen (Ziegler, Dufour, et al., 2024). Cleavage of protein phosphatase 1 regulatory subunit 1B (Ppp1r1b) at ^90^Asn↓^91^Leu was also detected in the inflamed colon by both FAIMS and bRP (Figure 3d). Considering the majority of these altered N‐terminal peptides exhibited negligible alterations in their protein abundance, we concluded that these are most likely the result of proteolysis rather than protein expression changes (Figure S5). Many of the detected potential substrates were unique to one fractionation approach, highlighting the value of multiplexing fractionation methods to more completely assess the protease degradome.

Considering the complementarity observed at the N‐terminomic level between FAIMS and bRP, we reasoned that conventional N‐termini enrichment may reveal additional legumain substrates. Utilizing HUNTER (Weng et al., 2019), we identified a total of 385 N‐terminal peptides (Table S5), including 168 N‐termini previously detected in both bRP and FAIMS fractionated samples, and a further 94 that were not observed using either bRP or FAIMS (Figure S6c). In line with our bRP/FAIMS analysis, several alterations in protease cleavage events were observed across naïve colons, with 17 N‐termini upregulated and nine downregulated (Figure S6a), while 13 WT‐enriched and nine *Lgmn* ^*−/−*^‐enriched N‐termini were detected in DSS‐treated colons (Figure S6b). Filtering for asparaginyl cleavages enriched in WT colons revealed 13 putative legumain substrates altered within the naïve colon and 11 in the inflamed colon (Figure S6a, b). Furthermore, of the putative legumain substrates identified using HUNTER, five in both naïve and inflamed conditions were unique to the HUNTER approach. These included asparaginyl cleavage of myosin‐11 (Myh11, O08638), La‐related protein 1 (Larp1, Q6ZQ58), heterogeneous nuclear ribonucleoprotein K (Hnrnpk, P61979), and tubulin alpha‐1B chain (Tuba1b, P05213), which were identified in both healthy and inflamed colons (Figure S6d, e). Peptidyl‐prolyl cis‐trans isomerase F (Ppif, Q99KR7) was also detected in naïve colons, and basement membrane‐specific heparan sulfate proteoglycan core protein (Hspg2, Q05793) was identified in the DSS colons. Together, these data expand our knowledge on the current repertoire of physiological legumain substrates by applying complementary degradomics approaches and generate a catalogue of novel cleavage sites in the naïve and inflamed murine colon (Table S6).

### 2.4 Bioinformatic analysis of putative legumain substrates indicates roles in actin organization and cellular junctions

**FIGURE 4:**
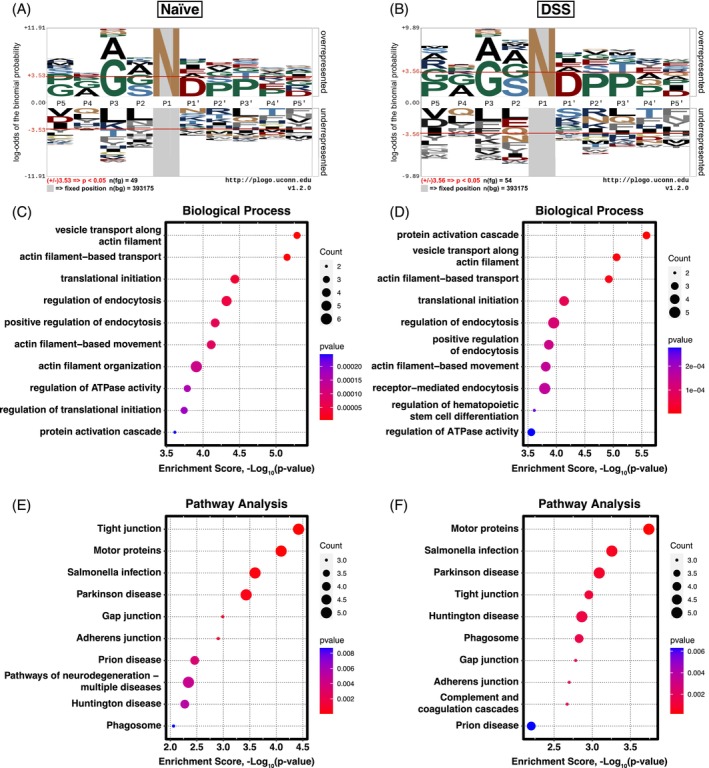
Gene ontology analysis of putative legumain substrates identified in naïve and inflamed (DSS) colon tissue by fractionated N‐terminomics approaches. N‐termini that were significantly enriched in the wild‐type (WT) colon and resulted from asparaginyl cleavage were considered to be putative legumain substrates. (a), (b) The genes for these N‐termini were processed in TopFINDer and pLogo to generate a sequence logo (https://plogo.uconn.edu/) for substrates identified in the (a) naïve (*n* = 49) and (b) inflamed (*n* = 54) colon. Overrepresented amino acids appear above and underrepresented below the *x*‐axis (*p* <0.05). (c)–(f) SRplot (https://www.bioinformatics.com.cn/en) was used to process the putative legumain substrates for gene ontology (GO) analysis and plotting. (c), (d) Enrichment of GO: Biological processes terms for naïve (c) and DSS (d) legumain substrates. (e), (f) KEGG pathways analysis for naïve (e) and DSS (f) legumain substrates.

Following the identification of putative legumain substrates using bRP, FAIMS, and HUNTER, we collated the list of reported cleavage sites in both the naïve and inflamed colon to assess cleavage site specificity. In total, the list of 62 N‐terminal peptides in the naïve colon originated from 47 different proteins, whereas the 70 N‐termini in the DSS colons corresponded to 55 proteins. The proteolytic cleavage specificity was similar in the naïve and inflamed colon (Figure 4a, b). Following enrichment of asparagine (N) in the P1 position, glycine (G) was favored in the P3 position, as well as a trending observation of aspartic acid (D) and proline (P) from P' to P3'. Compared to the previous analysis using the FAIMS data alone (Ziegler, Anderson, et al., 2024), this dataset, which is supplemented with results obtained using bRP and HUNTER, offers a more defined cleavage motif for legumain protease activity with enrichment of these amino acids in the P3 and P' to P3' positions.

We also examined enrichment of gene ontology (GO) terms using SRplot (https://www.bioinformatics.com.cn/en). Key terms included a range of cytoskeletal and endocytosis‐related biological processes, suggesting a potential role for legumain in regulating cell morphology and structure (Figure 4c, d). The protein activation cascade was highlighted as the most enriched biological process in the inflamed colon (Figure 4d). This included proteins such as fibulin‐1 (Fbln1, Q08879), fibrinogen (Fga, E9PV24), and coagulation factor XIII A chain (F13a1, Q8BH61), which may be an indicator of increased proteolysis in experimental colitis and activation of the coagulation cascade, which is often associated with ulcerative colitis. Pathway analysis also highlighted that legumain protease activity corresponds to cell junction regulation and neurodegenerative pathways (Figure 4e,f), which correlates with its known contribution to pathologies such as Alzheimer's and Parkinson's disease (Kang et al., 2022; Zhang et al., 2017). Changes in cleavages of proteins in the tight junction, adherens junction, and gap junction pathways may also associate legumain cleavage events to gut barrier function, which is impaired in IBD. Overall, these data shed light on the proteolytic contribution of legumain to normal colon function, and how dysregulation of legumain protease activity may contribute to DSS‐induced colitis in mice. Moreover, the combination of techniques to generate a more complete dataset allowed a more comprehensive and validated analysis of the potential role of legumain in the gut.

## 3 DISCUSSION

In this study, we demonstrated the utility of fractionation‐based, enrichment‐free approaches for the quantification of proteins and N‐terminal peptides in physiological samples. By comparing our previously reported N‐terminomics results obtained following online FAIMS fractionation (Ziegler, Anderson, et al., 2024) to offline bRP fractionation, we demonstrated that the detection of sub‐stoichiometric peptides such as N‐termini can be effectively quantified, with each approach detecting a unique subset of putative legumain substrates.

This work establishes bRP fractionation as an effective tool to improve proteome coverage and facilitate the detection of protease cleavage events in multiplexed biological samples as seen in previous studies with other post‐translational modifications (Batth et al., 2014; Wang, Kavdia, et al., 2020). Although bRP has previously been used for analysis of enriched N‐termini following either HUNTER or TAILS protocols (Kalogeropoulos et al., 2023; Meyer et al., 2021), this is the first time, to our knowledge, that it has been applied to an enrichment‐free method to assess the N‐terminome of physiological samples.

Previous studies have detected distinct peptide and protein identifications acquired by different fractionation methods in parallel (Cao et al., 2012). For example, Zheng et al. reported differences in the peptides and proteins quantified using either SDS‐PAGE or bRP prior to LC–MS/MS analysis (Zheng et al., 2022). This was also observed in the detection of human plasma and yeast proteomes (Deng et al., 2021; Zhao et al., 2016). Since each approach leverages different mechanisms of fractionation (Manadas et al., 2010), applying additional fractionation methods such as strong‐cation exchange or size‐exclusion chromatography (Vogele et al., 2024; Wang, Zhang, et al., 2021) may lead to the detection of additional exclusive peptides and N‐termini, which would further extend proteome coverage (Mostovenko et al., 2013).

In addition to fractionation, we also applied enrichment‐based N‐terminomics leveraging HUNTER to enable the discovery of an additional 94 cleavage sites not detected using either FAIMS or bRP (Figure S6). Of these, there were five N‐termini significantly enriched in the WT colon in both naïve and inflamed conditions that resulted from asparaginyl cleavage and therefore indicate putative legumain substrates solely identified in the HUNTER workflow. It has been well established that different N‐terminomics enrichment methods can lead to complementary results and improve the overall depth of the proteome (Hanna et al., 2023). Comparison of N‐ and C‐termini identified with various versions of the CHromatographic AMplification of Protein N‐terminal peptides (CHAMP) strategy has also indicated stark differences in their identifications between each workflow (Morikawa et al., 2024). This specifically highlights the importance of interrogating the C‐terminome to unveil further cleavage sites and validate the observed N‐termini.

Through proteomics analysis, we observed changes in protein abundance between WT and legumain‐deficient (*Lgmn* ^*−/−*^) mice in both naïve and inflamed colons (Figure 2c, d). Of the proteins identified, calpain‐9 was enriched in the *Lgmn* ^*−/−*^ colon under naïve conditions and detected following both FAIMS and bRP fractionation (Figure 2c; Table S2). Calpain‐9 is a calcium‐dependent cysteine protease that is highly expressed in the stomach and surrounding gastrointestinal tract (GIT) (Sorimachi et al., 1993). In the setting of gastric cancer, calpain‐9 is often downregulated and is speculated to play a protective role in the mucosal layer of the GIT (Peng et al., 2016). Overexpressing calpain‐9 also reduces cell viability by arresting cell cycle progression. Similarly, calcium‐activated chloride channel regulator 1 (Clca1) is consistently upregulated in naïve *Lgmn* ^*−/−*^ colon tissue (Figure 2c; Table S2) and has been identified as a tumor suppressor in colorectal cancer (Li et al., 2017). Considering this, the upregulation of these two proteins may offer a potential protective role in the absence of legumain. While legumain is associated with increased metastatic potential and worse patient outcomes in both colorectal and gastric cancers (Haugen et al., 2015; Wang, Zhang, et al., 2020), the direct association of legumain‐mediated effects in this context warrants further investigation. We also observed increases in multiple hemoglobin subunits solely in the inflamed colon in the presence of legumain (Figure 2d). Hemoglobin is used as a fecal marker for colonic inflammation in inflammatory bowel diseases (Mooiweer et al., 2014). Other known serum markers such as alpha‐1 anti‐trypsin (Serpina1d and Serpina1e) are also upregulated in inflamed WT colons (Cioffi, 2015). Together, these may indicate more severe inflammatory conditions in the presence of legumain. When combined with the previously reported changes identified using FAIMS‐facilitated N‐terminomics, this study provides the most comprehensive list of legumain‐dependent proteome changes in the gut, highlighting its contribution to both normal gut physiology and inflammatory bowel diseases (Table S6).

For N‐terminomics analysis, we observed a total of 5401 N‐termini following bRP‐fractionation, which is a~62% increase from the 3334 N‐termini using FAIMS‐facilitated N‐terminomics (Ziegler, Anderson, et al., 2024). Of these, we identified 17 putative legumain substrates in the naïve column, five of which were previously observed with FAIMS. Cleavage of lamina‐associated polypeptide 2 (Tmpo) at ^58^Asn↓^59^Ser was previously identified in naïve murine spleen and validated in vitro (Ziegler, Dufour, et al., 2024). We now show that legumain also cleaves Tmpo in the naïve colon, indicating broader implications of this proteolytic event. We also observed 48 asparaginyl cleavages enriched in the inflamed WT colon, five of which appear in both datasets (Figure 3d). Cleavage of protein phosphatase regulatory subunit 1B (Ppp1r1b) at ^90^Asn↓^91^Leu is the only one unique to the inflamed colon (Figure 3b; Table S4) (Log_2_(WT DSS/*Lgmn* ^*−/−*^ DSS) = 2.996 and ‐log_10_(*p*‐value) = 1.434 in bRP‐fractionated samples). Ppp1r1b regulates the activity of protein‐phosphatase 1 (PP1), which removes phosphate groups from various proteins (Hemmings et al., 1984). Upregulation of Ppp1r1b is associated with gastric, breast, colon, esophageal, lung, and prostate cancers (Avanes et al., 2019), and correlates to worse survival outcomes in pancreatic cancer (Tiwari et al., 2020). Apart from legumain, calpains can cleave Pppr1r1b at ^153^Thr↓^154^Cys in humans (^147^Thr↓^148^Cys in mice) in neurodegeneration. This cleavage event contributes to increased PP1 activity and reduced phosphorylation of cAMP‐responsive element‐binding protein (CREB) (Cho et al., 2015), subsequently increasing plaque deposition and worsening symptoms of Alzheimer's disease. While the impact of Ppp1r1b cleavage in the gut is unknown, it is possible that legumain‐mediated cleavage of Pppr1r1b contributes to colitis pathogenesis.

Our dataset also revealed N‐termini not directly dependent on legumain activity, but instead on other proteases, which may exhibit altered activity in the presence or absence of legumain (Figure 3a, b). We observed increased abundance of trypsin‐like protease inhibitors Serpina1d and Serpina1e, especially in the inflamed colon, suggesting a decrease in the proteolytic activity of these proteases in the WT colon (Figure 2c, d). Our N‐terminomics analysis also revealed a range of cleavage events which suggest increased activity of proteases such as cathepsin X in the naïve and inflamed WT colon, and cathepsins B and L in the *Lgmn* ^*−/−*^ colon, the latter of which most likely corresponds to a compensatory increase in the abundance and subsequent activation of these lysosomal proteases in the absence of legumain. As such, these cathepsins may exhibit altered proteolytic processing abilities and contribute to the repertoire of cleavage events identified. The changes to protease activation and their subsequent substrates in these conditions may offer further routes to understanding gut function and warrant further investigation.

Herein, we have demonstrated the utility of complementary fractionation approaches for the increased identification of peptides and proteins in biological samples. By improving the proteome coverage, less abundant and sub‐stoichiometric peptides such as N‐termini can be observed without the need for prior N‐terminal peptide enrichment. We show that bRP fractionation offers an expanded set of peptide quantifications that are unique to FAIMS‐fractionated analysis. We have identified further legumain‐mediated proteome and N‐terminome changes within the murine gut, which expands our understanding of its proteolytic role in both the naïve and inflamed colon. This study not only provides a deeper understanding of the role of legumain but also reveals the value of using multiple N‐terminomics approaches to assess the degradome.

## 4 MATERIALS AND METHODS

### 4.1 Mice

All studies involving mice were approved by and performed in accordance with the Animal Ethics Committee guidelines established at the New York University Institutional Animal Care and Use Committee. WT (C57BI6 J) mice were sourced from JAX (#000664) and legumain‐deficient mice (Matthews et al., 2010) were a gift from Thomas Reinheckel. All mice were maintained under controlled temperature and light conditions with free access to food and water.

Experimental acute colitis was induced in mice (8–10 weeks old) over 6 days by administering 3% DSS, (MP Biomedicals 36,000–50,000 Da) in the drinking water. As a control, a group of mice also received normal drinking water (naïve). After 6 days, the mice were sacrificed, and the colons were harvested and flushed with phosphate buffered saline. The colon tissue was kept frozen at −80°C until N‐terminomic analyses.

### 4.2 Collection and labeling of protein lysates for N‐terminomics analysis

Tissue lysis and N‐terminal labeling were carried out as previously described (Ziegler, Anderson, et al., 2024). Briefly, vehicle and DSS colons from WT and legumain‐deficient (*Lgmn* ^*−/−*^) mice were homogenized in 4% SDS lysis buffer (4% SDS, 50 mM HEPES, pH 7.5 (Sigma)) containing Roche cOmplete EDTA‐free protease inhibitor (Sigma) using a probe sonicator. Lysates were cleared by centrifugation (21,000 × *g*, 5 min, 4°C) before normalization using BCA analysis, where samples were aliquoted to contain 20 μg protein in 100 μL lysis buffer for subsequent labeling.

Proteins were reduced using 20 mM DTT (10 min, 80°C, 500 rpm) followed by alkylation with 50 mM iodoacetamide (30 min, 37°C, 500 rpm) and quenching with a further 50 mM DTT (20 min, 37°C, 500 rpm). Proteins were precipitated using excess conditioned SP3 paramagnetic beads (Cytiva Sera‐Mag SpeedBeads 45152105050250 and 65152105050250, 1:40 protein:bead ratio) with the addition of 100% ethanol (80% final) for 20 min with gentle agitation (25°C, 1000 rpm) (Hughes et al., 2019). The peptide‐conjugated beads were washed three times with 80% ethanol facilitated by a magnetic stand prior to primary amine labeling using TMTpro 16‐plex (Thermo, #A44520). Peptides underwent two stages of labeling with 80 μg TMTpro reagent resuspended in 10 μL 100% acetonitrile for 60 min each time (25°C, 1000 rpm) (Zecha et al., 2019). Labeling was quenched with the addition of 3% hydroxylamine (2 μL, Merck, #438227) under the same conditions. Peptides were again precipitated using conditioned SP3 beads (1:20 protein:bead ratio) and 100% ethanol (80% final) for 20 min (25°C, 1000 rpm), and then washed in 80% ethanol three times using a magnetic stand. Peptide‐conjugated beads were resuspended in 100 μL 200 mM HEPES (pH 7.5) containing Solu‐trypsin (1 μg, Sigma, 1:20 trypsin:protein ratio) for an overnight digestion at 37°C (1,000 rpm). Following this, the peptides were collected, pooled, and acidified using Buffer A* (0.1% trifluoroacetic acid, 2% ACN) for de‐salting using a 50 mg Sep‐Pak Cartridge C18 Column (Waters, WAT054960) (Demir et al., 2017). Samples were dried using a speedvac and stored at −20°C until mass spectrometric analyses.

### 4.3 Negative selection by high‐efficiency undecanal‐assisted N‐termini EnRichment (HUNTER)

HUNTER was completed according to the protocol of Weng and colleagues (Weng et al., 2019). Briefly, labeled peptides were resuspended in 100 mM triethylammonium bicarbonate pH 8.0, and undecanal solution (Sigma, #80139) stock was prepared by diluting to 40% (v/v) in 100% acetonitrile. Undecanal solution was added to samples at a 50:1 undecanal:peptide ratio along with 30 mM sodium cyanoborohydride (Sigma, #156159), with the pH confirmed to be between 7.0 and 8.0. Labeling was allowed to proceed for 90 min at 50°C (1,000 rpm) and then samples were adjusted to 40% ethanol and 0.5% trifluoroacetic acid (TFA). Negative selection was performed using a 50 mg Sep‐Pak Cartridge C18 Columns (Waters, WAT054960). Columns were conditioned using 100% methanol, followed by 40% ACN + 0.1% TFA before samples were loaded. The resulting flow‐through was collected, and the columns were washed using 40% ethanol +0.1% TFA for further N‐termini collection. HUNTER samples were dried on a speedvac and stored at −20°C prior to analysis.

### 4.4 Chromatographic separation of peptides by high pH (basic) reverse‐phase fractionation

Basic reverse‐phase (bRP) fractionation was performed on a Dionex 3500 equipped with a UV detector. Twelve μg of labeled peptide was injected onto a C18BEH 0.3 mm * 150 mm column containing 1.7 μm particles (Waters) at a flow rate of 5 μL/min. Separation was achieved using a 50‐min gradient of 5%–40% Buffer B (Buffer A = 10 mM ammonium formate, pH 7.9; Buffer B = 90% acetonitrile). A total of 48 fractions were collected and automatically concatenated into 12 fractions in a looping fashion directly into a 96‐well plate. Samples were dried in a speedvac and stored at −20°C prior to analysis.

### 4.5 High‐FAIMS and liquid chromatography tandem mass spectrometry (LC–MS/MS) analysis

Peptides were separated using a two‐column chromatography setup composed of a PepMap100 C18 20‐mm by 75‐μm trap and a PepMap C18 500‐mm by 75‐μm analytical column (Thermo Fisher Scientific) on a Dionex Ultimate 3000 UPLC (Thermo Fisher Scientific). Samples were concentrated onto the trap column at 5 μL/min for 5 min with Buffer A (0.1% formic acid, 2% DMSO) and then infused into an Orbitrap Fusion Lumos mass spectrometer (Thermo Fisher Scientific) equipped with a FAIMS Pro interface at 300 nL/min. For FAIMS‐facilitated N‐terminomics, fractionation occurred as previously described (Ziegler, Anderson, et al., 2024), injecting 2 μg of peptides per fraction over six compensational voltages (CV) of −20, −30, −40, −50, −60, or −70. Basic reverse‐phase fractionated, pre‐HUNTER, and HUNTER samples were analyzed as above without the use of FAIMS.

Each sample was separated using 125‐min analytical runs undertaken by altering the buffer composition from 3% Buffer B (0.1% formic acid, 77.9% acetonitrile, 2% DMSO) to 23% B over 95 min, then from 23% B to 40% B over 10 min, then from 40% B to 80% B over 5 min. The composition was held at 80% B for 5 min, and then dropped to 3% B over 0.1 min before being held at 3% B for another 9.9 min. Data‐dependent acquisition was undertaken with a single Orbitrap MS scan (300–2000 *m*/*z*, a resolution of 60k with the automated gain control (AGC) set to a maximum of 400%) collected every 3 s followed by Orbitrap MS/MS HCD scans of precursors (Quad isolation window width of 1.6 *m*/*z*, stepped normalized collision energy of 35;38;45%, maximal injection time of 118 ms, a resolution of 60k and a AGC of 500%, lower mass cut off set at 120 *m*/*z*).

### 4.6 Bioinformatic analysis

Mass spectrometry raw data files were matched to a murine proteome database containing isoforms and contaminants (UniProt Accession: UP000000589, accessed January 2024, contains 51,316 entries including 25,658 decoys = 50.0%) using FragPipe v.21.1 (MSFragger v.4.0) (Kong et al., 2017). For fractionation‐based experiments, all fractions of a biological sample were defined as a single replicate, and experiments were searched together for a global false discovery rate (FDR) of 1% (Schaab et al., 2012). Variable modifications were set to include methionine oxidation (+15.9949 Da), N‐terminal acetylation (+42.0106 Da), N‐terminal cyclisation on glutamine (−17.026549 Da) and glutamic acid (−18.010565 Da), N‐terminal and lysine TMTpro‐labeling (+304.20715 Da), and N‐terminal lysine labeling (+608.4143 Da). A fixed modification of cysteine carbamidomethylation (+57.0215 Da) was also included. Cleavage specificity was set to “SEMI‐N_TERM” and “TrypsinR” (Arg‐C) with a maximum of two missed cleavages allowed. Mass tolerances for precursor and fragment ions were set to 20 ppm. An isotopic error was set to 3 Da. FDR was determined using Philosopher (v.5.1.0) and set at both the protein and peptide levels. Isobaric quantification of TMTpro‐labeled peptides was performed using IonQuant (v.1.10.12) (Yu et al., 2021), with a quantification level of 2, mass tolerance of 20 ppm, and a virtual reference selected as no pooled sample was included.

Further filtering and statistical analyses of the normalized protein and peptide intensity values were processed in Perseus (v.1.6.0.7) (Tyanova et al., 2016). Values were log_2_‐transformed and filtered to contain a minimum of three of four valid values in at least one of the four groups. The remaining missing values were imputed based on a normal distribution (*σ*‐width = 0.3 and *σ*‐downshift = 1.8) before a two‐sample t‐test was applied to calculate fold‐change and *p*‐value, which included default permutation‐based multiple‐hypothesis correction. Data visualization was performed using R (v.4.3.1). GO and pathway analyses were performed using SRplot (Tang et al., 2023). TopFINDer was used to extract upstream peptide sequences (Fortelny et al., 2015) for the generation of sequence logos using pLogo (O'Shea et al., 2013).

### 4.7 Statistical analysis

All experiments were performed with four biological replicates per group. The significance threshold for N‐terminomics analysis was set to log_2_(fold‐change) ± 1 and ‐log_10_(p‐value) = 1.3 (p‐value = 0.05).

## AUTHOR CONTRIBUTIONS

**Alexander R. Ziegler:** Conceptualization; investigation; writing – original draft; methodology; validation; visualization; formal analysis; data curation. **Benjamin L. Parker:** Methodology; resources; writing – review and editing; investigation. **Nichollas E. Scott:** Conceptualization; investigation; funding acquisition; writing – review and editing; methodology; supervision; resources. **Laura E. Edgington‐Mitchell:** Conceptualization; funding acquisition; writing – review and editing; project administration; supervision; resources.

## CONFLICT OF INTEREST STATEMENT

There authors report no conflicts of interest.

## Supporting information


**Figure S1.** Fractionation and N‐terminomics approaches to assess the proteolytic activity of legumain in murine colon.
**Figure S2.** TMTpro labeling efficacy in multiplexed FAIMS and bRP fractionated samples.
**Figure S3.** Peptide properties of all peptides identified fractionation‐based N‐terminomics approaches.
**Figure S4.** Peptide properties of all N‐termini identified fractionation‐based N‐terminomics approaches.
**Figure S5.** Legumain‐dependent cleavage events are due to proteolysis rather than protein abundance changes.
**Figure S6.** Conventional negative selection of N‐termini using High‐efficiency Undecanal‐based N‐Termini EnRichment (HUNTER).

**Table S1.** Protein quantifications and statistics between wild‐type (WT) and legumain‐deficient (Lgmn^−/−^) naïve and DSS colon tissue analyzed by basic reverse‐phase fractionation.
**Table S2.** Protein changes between wild‐type (WT) and legumain‐deficient (*Lgmn* ^*−/−*^) naïve and DSS colon tissue analyzed by bRP and FAIMS.
**Table S3.** N‐termini quantifications and statistics between wild‐type (WT) and legumain‐deficient (*Lgmn* ^*−/−*^) naïve and DSS colon tissue analyzed by basic reverse‐phase fractionation.
**Table S4.** N‐termini changes between wild‐type (WT) and legumain‐deficient (*Lgmn* ^*−/−*^) naïve colon tissue analyzed by bRP and FAIMS.
**Table S5.** N‐termini quantifications and statistics between wild‐type (WT) and legumain‐deficient (*Lgmn* ^*−/−*^) naïve and DSS colon tissue identified in HUNTER samples.
**Table S6.** Putative legumain substrates identified across naïve and inflamed colon tissue and naïve spleen tissue from wild‐type (WT) and legumain‐deficient (*Lgmn* ^*−/−*^) mice.

## Data Availability

The mass spectrometry proteomics data have been deposited in the Proteome Xchange Consortium via the PRIDE partner repository (Perez‐Riverol et al., 2022) with the following data set identifiers: PXD051470 (FAIMS‐fractionated data), PXD060852 (bRP‐fractionated data) and PXD060854 (HUNTER data).
