# Supplementary material for: Employing complementary fractionation‐based N‐terminomics approaches enhances the identification of legumain cleavage events in naïve and inflamed colon tissue
Source: Protein Sci. 2025 Sep 19;34(10):e70186. doi: 10.1002/pro.70186 (PMC12447244; doi:10.1002/pro.70186)
Supplement: Supplementary file 1 — Figure S1. Fractionation and N‐terminomics approaches to assess the proteolytic activity of legumain in murine colon. Figure S2. TMTpro labeling efficacy in multiplexed FAIMS and bRP fractionated samples. Figure S3. Peptide properties of all peptides identified fractionation‐based N‐terminomics approaches. Figure S4. Peptide properties of all N‐termini identified fractionation‐based N‐terminomics approaches. Figure S5. Legumain‐dependent cleavage events are due to proteolysis rather than protein abundance changes. Figure S6. Conventional negative selection of N‐termini using High‐efficiency Undecanal‐based N‐Termini EnRichment (HUNTER). [file PRO-34-e70186-s001.pdf]

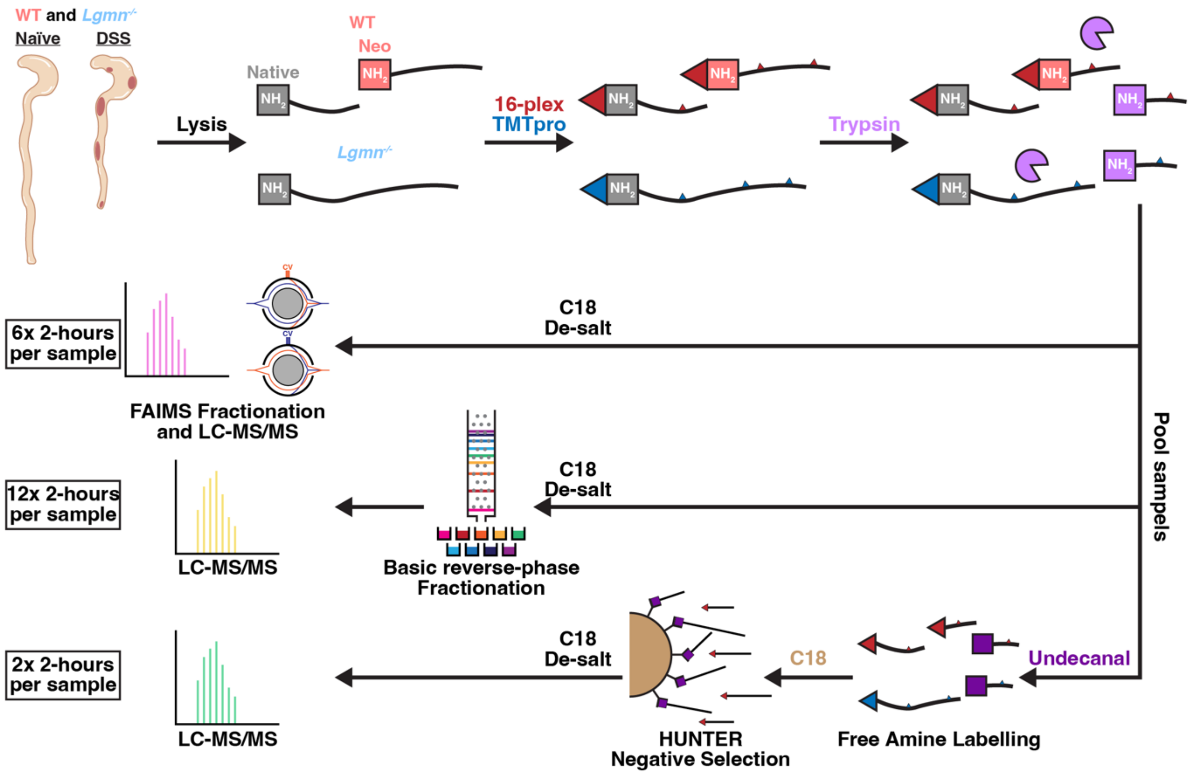

Fig. S1. Fractionation and N-terminomics approaches to assess the proteolytic activity of legumain in murine colon. Colon tissue was harvested from naïve or DSS-treated wild-type (WT) and legumain-deficient (*Lgmn*$^{-/-}$) mice (n = 4/group). Tissue was lysed and peptides were reduced and alkylated prior to primary amine labelling of N-termini and lysine side chains with 16-plex TMTpro. Peptides were digested with trypsin, de-salted with C18 and subjected to fractionation by either high-field asymmetric waveform ion mobility spectrometry (FAIMS) or basic reverse-phase (BRP) chromatography prior to mass spectrometry analysis. For FAIMS fractionation, six fractions were collected by altering the compensation voltage (CV, -70, -60, -50, -40, -30, -20). For BRP fractionation, 48 fractions were collected over a one-hour gradient and concatenated into 12 fractions. Each fraction was run on an Orbitrap Lumos mass spectrometer over a two-hour gradient for proteomics and N-terminomics analyses (12 µg protein per experiment).

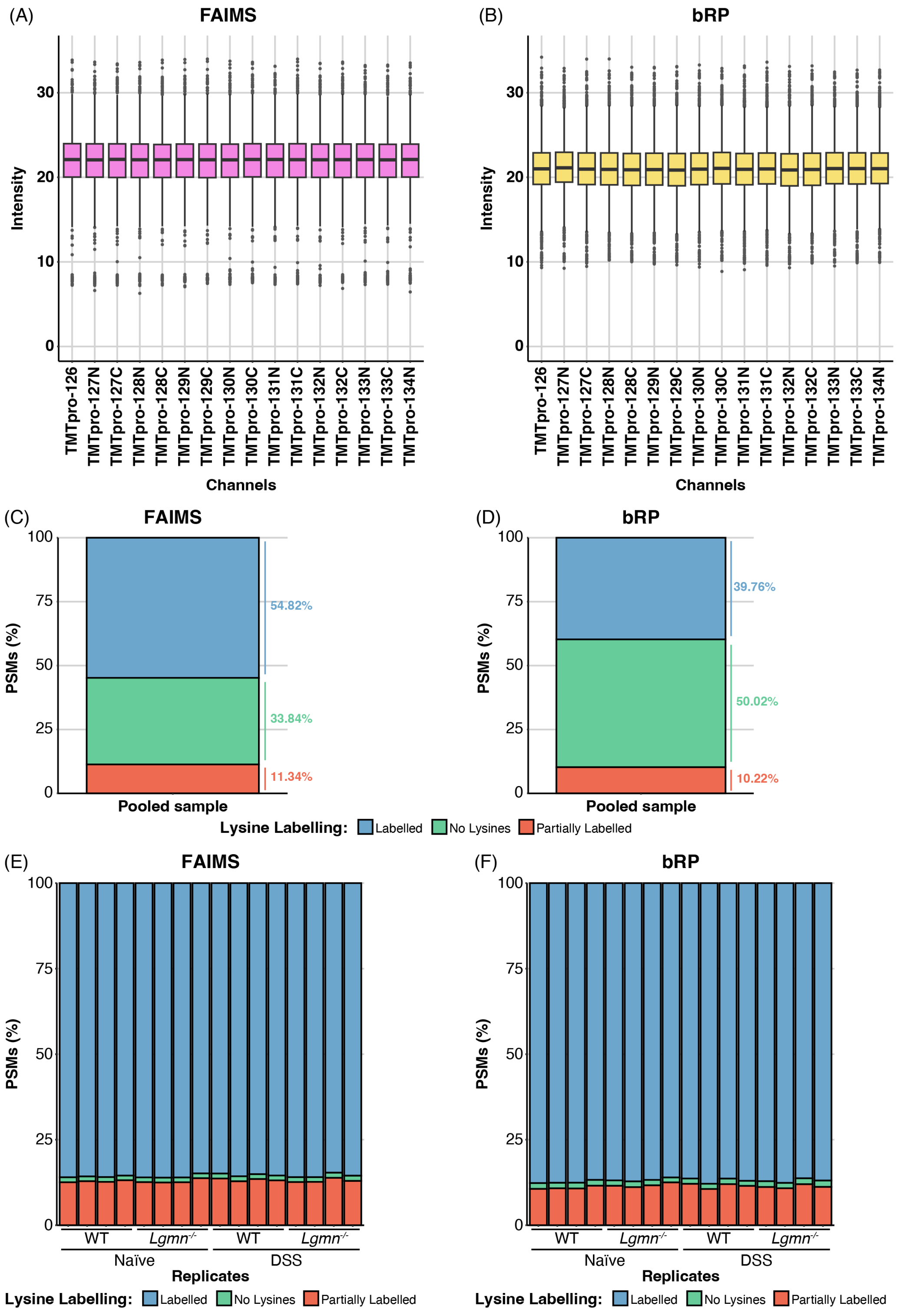

Fig. S2. TMTpro labelling efficacy in multiplexed FAIMS and bRP fractionated samples. (A-B) Boxplots of the average intensity of protein quantifications per TMTpro channel in FAIMS (A) and bRP (B) fractionated samples. Outliers are shown as grey dots. **(C-D).** Labelling efficacy was measured per peptide-spectrum match (PSM) using the internal lysines. Peptides with all lysines labelled are shown in blue, those with no lysines are in green, and those with incomplete lysine labelling in red. **(E-F).** Labelling efficacy was also performed per TMTpro channel/replicate.

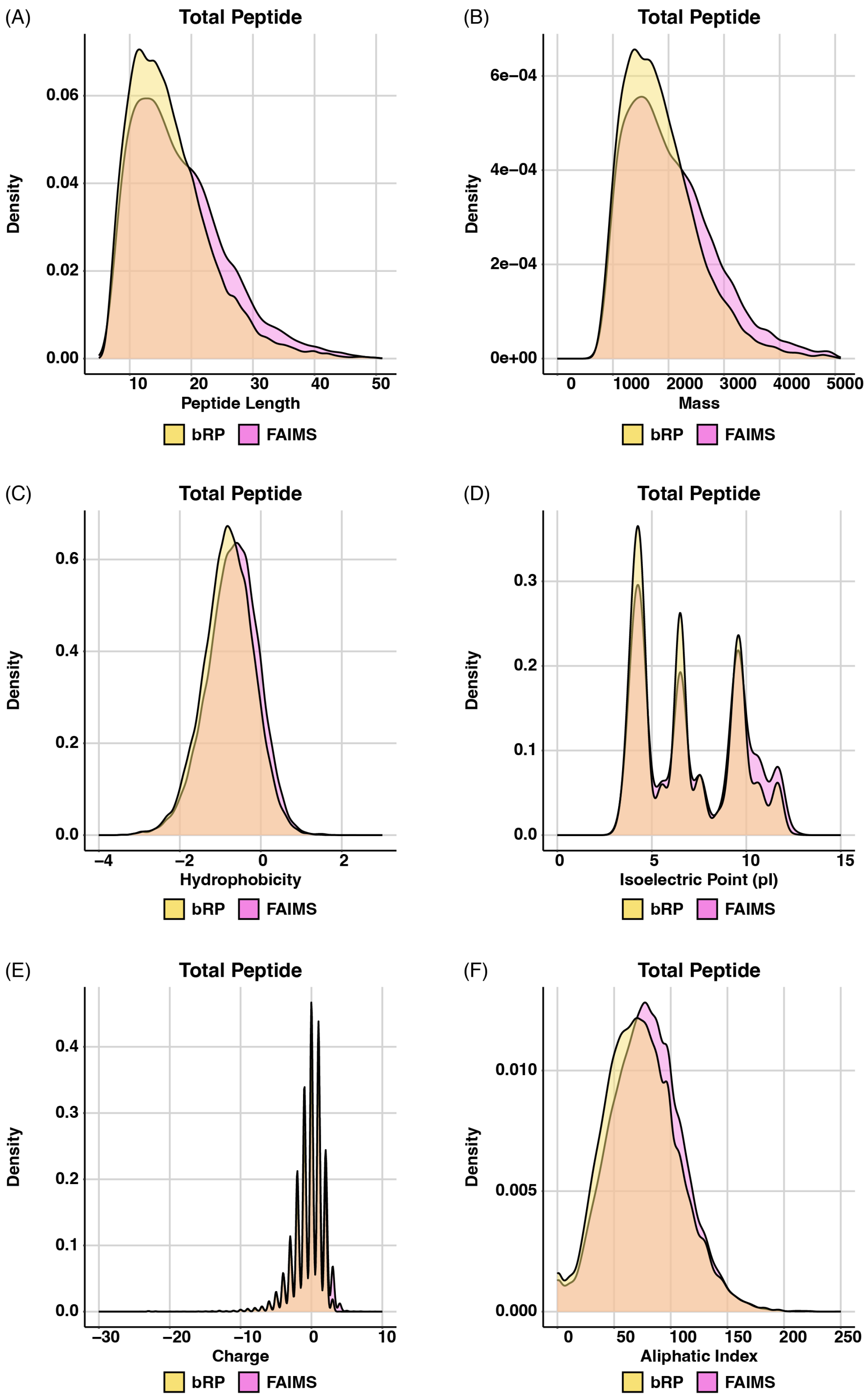

Fig. S3. Peptide properties of all peptides identified fractionation-based N-terminomics approaches. (A-F). All peptides quantified by FragPipe (v.22.0) across all biological replicates (n = 4/group) were analysed according to the indicated peptide property using the Peptide package (v.2.4.5) with default setting in R (v.4.4.2). Peptides quantified in the FAIMS fractionation workflow are shown in pink and those quantified in the BRP fractionation workflow in yellow.

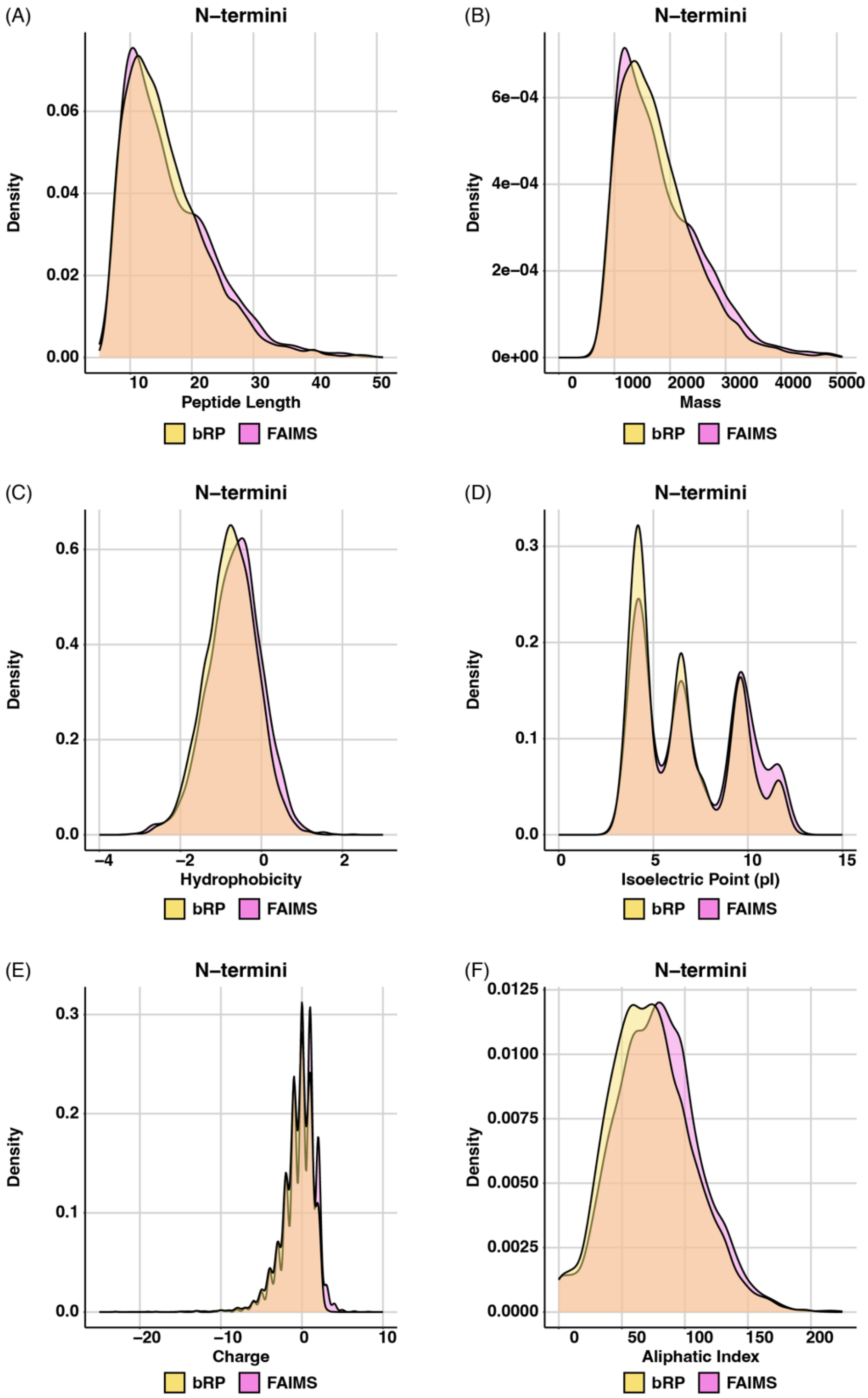

Fig. S4. Peptide properties of all N-termini identified fractionation-based N-terminomics approaches. (A-F). N-termini were filtered from the total peptide population using N-terminal TMTpro labelling as a tag. All N-termini quantified across all biological replicates (n = 4/group) were analysed according to the indicated peptide property using the Peptide package (v.2.4.5) with default setting in R (v.4.4.2). N-termini quantified in the FAIMS fractionation workflow are shown in pink and those quantified in the BRP fractionation workflow in yellow.

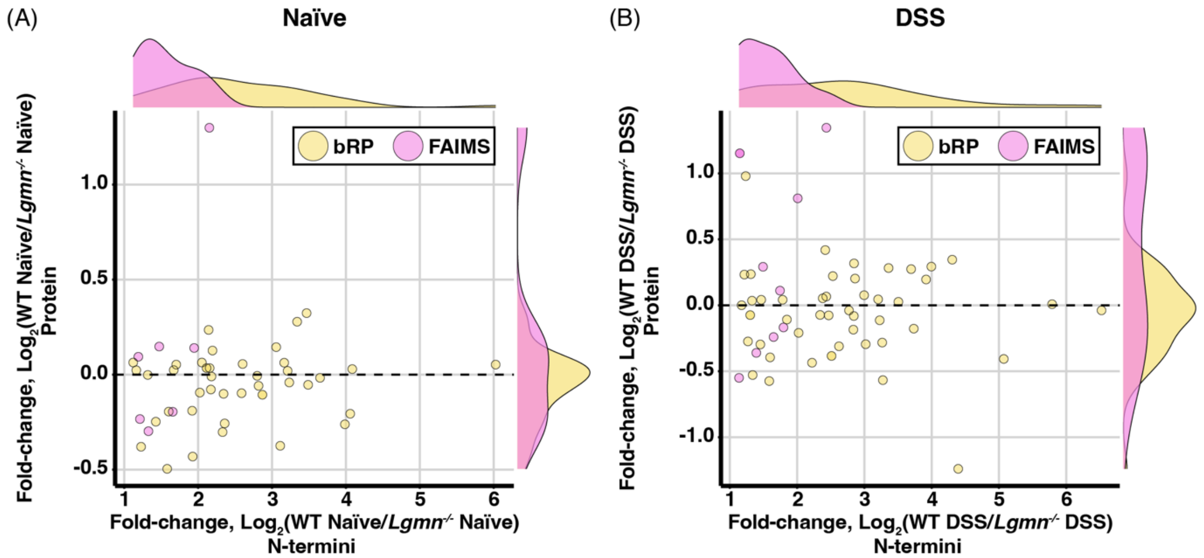

Fig. S5. Legumain-dependent cleavage events are due to proteolysis rather than protein abundance changes. (A-B) Scatter plots of the fold change of the cleavage event and its corresponding protein abundance changes with yellow points indicating substrates identified with bRP and pink with FAIMS in naïve (A) and DSS (B) mice. Density plots of the individual points are shown on the x and y axes.

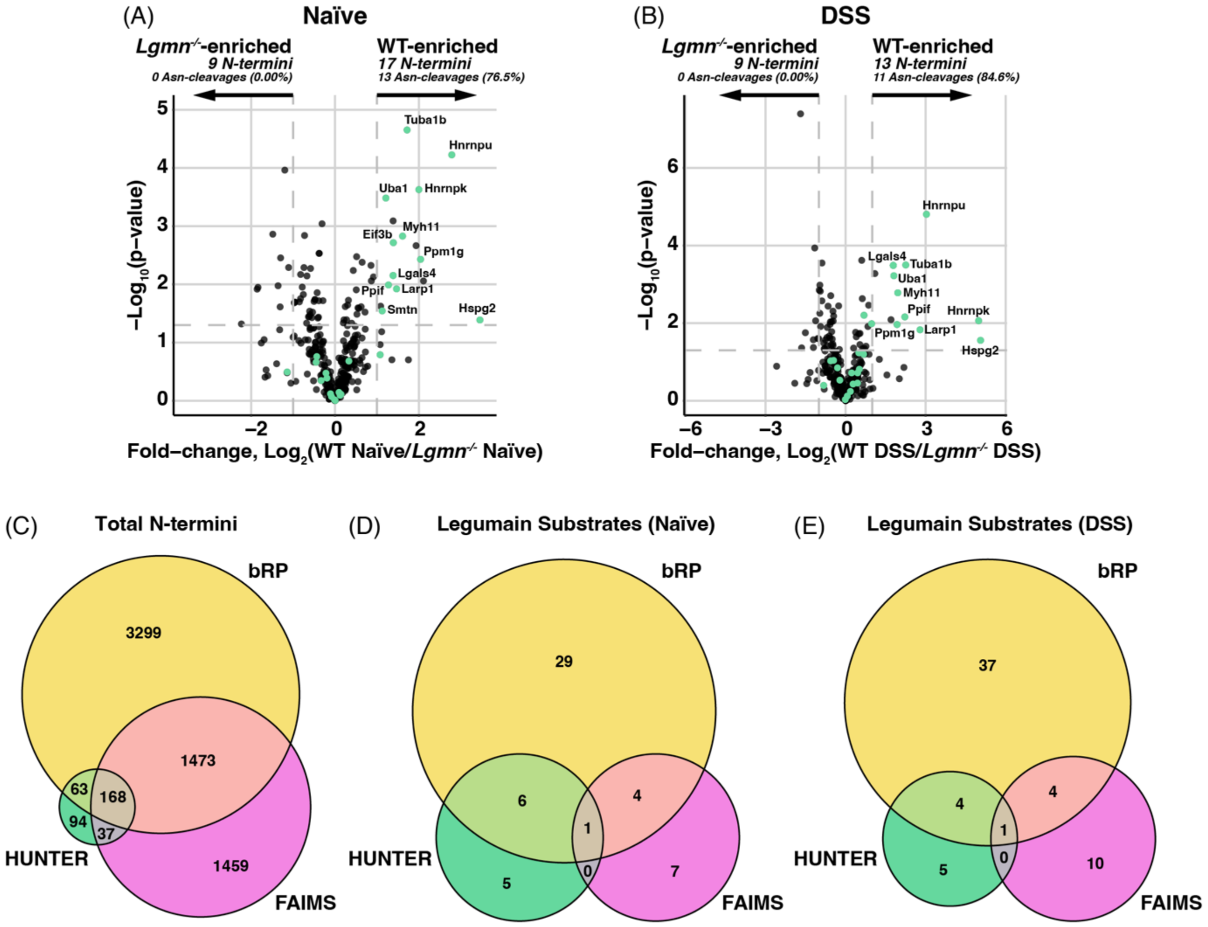

Fig. S6. Conventional negative selection of N-termini using High-efficiency Undecanal-based N-Termini EnRichment (HUNTER). Following sample de-salting, peptides were subject to undecanal labelling and negative selection of native and neo-N-termini using the HUNTER method. The resulting N-termini were analysed by mass spectrometry for N-terminomics analysis. **(A-B).** N-termini were filtered from the total peptide identification by filtering for N-terminal TMTpro labelling. N-termini identified in either naïve (A) or DSS-treated (B) colons were subjected to a student's two-sample t-test and visualised by volcano plot. A Log_2(WT/*Lgmn*$^{-/-}$) > |1| and -Log_{10}(p-value) > 1.3 were considered as statistically significant (n = 4/group). N-termini arising from cleavage following an asparagine residue are highlighted in green. **(C-E).** Overlap of the total N-termini (C), naïve legumain substrates (D), and DSS legumain substrates (E) identifications between HUNTER and the two fractionation-based N-terminomics workflows.
